# Supplementary material for: Tunable chiral and nematic states in the triple-Q antiferromagnet Co1/3TaS2
Source: Nat Commun. 2026 Jan 31;17:2212. doi: 10.1038/s41467-026-68843-0 (PMC12963503; doi:10.1038/s41467-026-68843-0)
Supplement: Supplementary file 1 — Supplementary Information [file 41467_2026_68843_MOESM1_ESM.pdf]

Supplementary Information: Tunable chiral and nematic states in the triple-Q antiferromagnet $Co_{1/3}TaS_2$

Erik Kirstein [1,*] Pyeongjae Park [2,3,*] Woonghee Cho [3]
Cristian D. Batista [4,5,†] Je-Geun Park [3,6,†] and Scott A. Crooker [1,†]

[1] National High Magnetic Field Laboratory, Los Alamos National Lab, Los Alamos, NM 87545, USA
[2] Materials Science and Technology Division, Oak Ridge National Laboratory, Oak Ridge, TN 37831, USA
[3] Department of Physics and Astronomy, Seoul National University, Seoul 08826, Korea
[4] Department of Physics and Astronomy, University of Tennessee, Knoxville, TN, USA
[5] Shull Wollan Center—A Joint Institute for Neutron Sciences, Oak Ridge National Laboratory, Oak Ridge, TN, USA
[6] Institute of Applied Physics, Seoul National University, Seoul 08826, Korea

1. SUPPLEMENTARY SECTION 1: EXPERIMENTAL DETAILS

A. Spectral dependence of MCD and MLD optical response

The magnitude and sign of the MCD signals that arise from the spin chirality of the noncoplanar triple-**Q** antiferromagnetism in $Co_{1/3}TaS_2$ depend on the wavelength λ – or equivalently, the photon energy – of the probe light. Figure S1a shows examples of MCD versus applied out-of-plane magnetic field H hysteresis loops, taken with probe light having different λ, at a fixed temperature T=15 K. The amplitude of the open hysteresis loop varies with λ, peaking at $\lambda \approx 700$ nm and inverting sign around $\lambda \approx 950$ nm. Moreover, the slope of the linear background is also λ-dependent. And finally, the magnitudes of the additional steps that appear at the metamagnetic transition at $H_m \approx \pm 3.5$ T also vary with λ (this latter aspect is discussed further in the next subsection, which considers offsets and artifacts in MCD measurements that can arise from linear dichroism).

Similarly, the MLD signals that arise from the single-**Q** (stripe-like) AFM order in $Co_{1/3}TaS_2$ are also λ-dependent, as can be seen from the examples of MLD(H) curves shown in Fig. S1b.

These various spectral dependencies were explicitly measured as a continuous function of probe wavelength λ, and are plotted in Figs. S1c-e as a function of the photon energy of the probe light. The amplitude of the open hysteresis loop in MCD studies was determined via the difference of two MCD(λ) spectra acquired at $H = +1.5$ T, one acquired after ramping H down from large positive value, and the other acquired after ramping H up from large negative value (thus, on the upper and lower branches of the hysteresis loop). The amplitude of the MLD signal was derived via the difference of two zero-field MLD(λ) spectra, one acquired at $T = 40$ K (in the nonmagnetic state) and the other acquired at $T = 27$ K (in the single-**Q** Phase III).

Each of these spectral dependencies is different and has its own "spectral fingerprint", as expected because each of these quantities derives from different combinations of the diagonal and off-diagonal complex conductivities $\sigma_{xx}(\omega)$, $\sigma_{yy}(\omega)$, and $\sigma_{xy}(\omega)$ at optical frequencies. In turn, these optical conductivities derive from the underlying spin order in $Co_{1/3}TaS_2$ and the influence of the spin order on the band structure and on the interband optical transitions occurring within this band structure. Given that spin-polarized bandstructures and frequency-dependent optical conductivities $\sigma(\omega)$ are routinely calculated for magnetic materials by a variety of first-principles theoretical methods (e.g., density functional theory), we anticipate that broadband magneto-optical measurements of MCD and MLD (and also closely-related effects such as Kerr rotation and Kerr ellipticity) can provide important benchmarks against which theoretical approaches can be compared and refined.

B. Cross-talk between measurements of circular and linear dichroism

These experiments use a commercial photoelastic modulator (PEM) from Hinds Instruments to modulate the polarization of the probe light between right- and left-circular for MCD studies (and between linear and cross-linear for MLD studies). In measurements of this type, it has long been known [1, 2] that the presence of any *linear* dichroism (or linear birefringence) in the sample, can generate small artifacts and offsets during measurements of *circular* dichroism (and vice-versa). In other words, changes in a material's linear dichroism (due, e.g., to the emergence of nematic order and concomitant anisotropy of the in-plane optical conductivity) can lead to offsets and artifacts in measurements of circular dichroism. In our measurements of $Co_{1/3}TaS_2$, signatures of these artifacts or "cross-talk" between circular dichroism and linear dichroism measurements are apparent in the unequal steps of the MCD(H) signals at the metamagnetic phase transition fields $H_m \approx \pm 3.5$ T. For $|H| > H_m$ there

* These authors contributed equally to this work.
† Correspondence: crooker@lanl.gov, jgpark10@snu.ac.kr, cbatist2@utk.edu

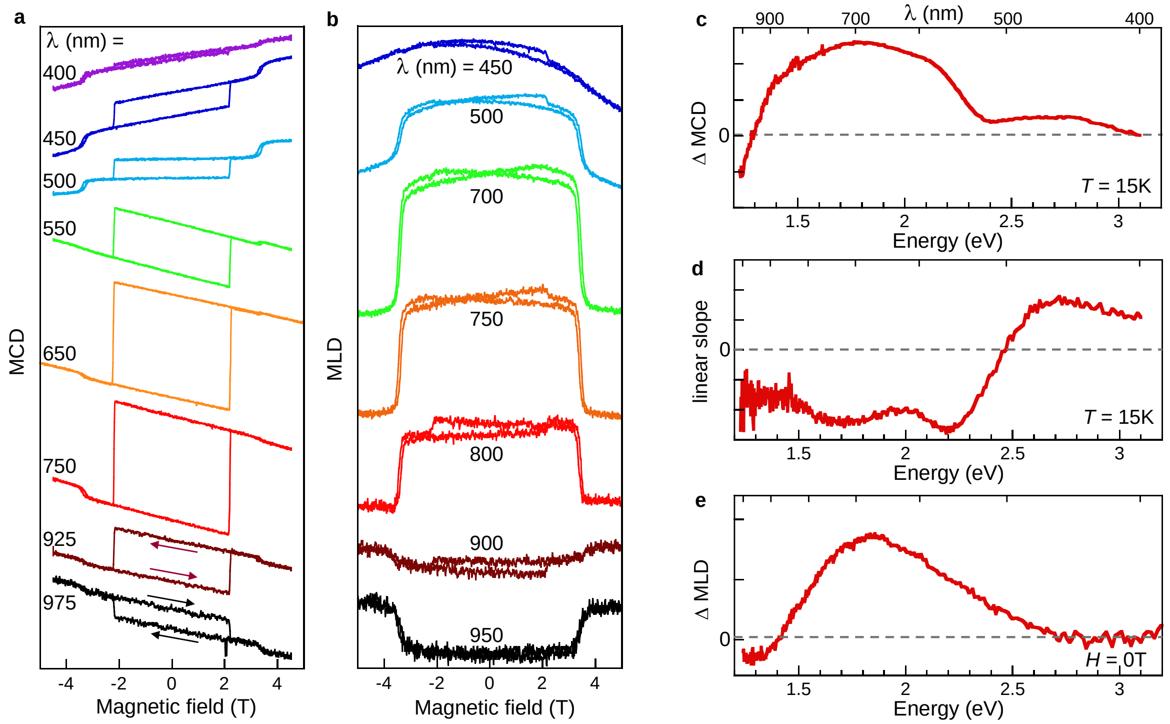

FIG. S1. **Dependence of MCD and MLD signals on the wavelength λ of the probe light.** **a**, Examples of MCD(H) hysteresis loops taken on $Co_{1/3}TaS_2$ at a fixed temperature (15 K), using probe light of different wavelengths (curves vertically offset for clarity). The amplitude (and sign) of the open hysteresis loop, the slope of the linear background, and the amplitude of the steps at the metamagnetic transition $H_m \approx \pm 3.5$ T, all vary with λ. **b**, Examples of MLD(H) measurements taken on $Co_{1/3}TaS_2$ at a fixed temperature (15 K), using probe light of different wavelengths. The magnitude and sign of the MLD signals that emerge for $|H| < H_m$ vary with λ. Note that a parabolic background is present at short λ. **c-e**, Explicit spectral dependencies, plotted as a continuous function of the probe light's photon energy, of **c**, the amplitude of the open hysteresis loop in MCD, **d**, the background linear slope of the MCD, and **e**, the amplitude of the MLD signal. These three spectral dependencies, which are not the same, derive from the interband optical transitions within the band structure, which are influenced by the underlying AFM spin order in $Co_{1/3}TaS_2$. These detailed spectral dependencies can be used to benchmark first-principles calculations of the diagonal and off-diagonal complex optical conductivities $\sigma_{xx}(\omega)$, $\sigma_{yy}(\omega)$, and $\sigma_{xy}(\omega)$ at optical frequencies.

is no linear dichroism (i.e., no nematic order), but for $|H| < H_m$ linear dichroism exists (because single-$\mathbf{Q}$ magnetism and nematicity are present), which causes a small offset in the MCD signal in this low-field range.

As shown pictorially in Fig. S2, this effect causes the steps in MCD at $+H_m$ and $-H_m$ to be unequal and no longer symmetric. As discussed in the previous subsection, the amount of cross-talk is wavelength-dependent, as can be seen from the various MCD(H) curves in Fig. S1a. Note that at wavelengths where the MLD signals due to nematicity are approximately zero (i.e., at $\lambda \approx$ 450 nm and 900 nm), the offsets vanish and MCD(H) traces have the expected symmetry – meaning that the steps in MCD at $\pm H_m$ are approximately equivalent.

Further, we emphasize that both the magnitude and sign of the cross-talk can also depend on sample location: If the probe beam interrogates a region of the sample containing nematic domains oriented primarily along (for example) 0°, then the offset due to cross-talk will have a particular sign (e.g., positive). But if the probe beam is moved to a different region of the sample where the nematic domains are oriented primarily along 120° or 240°, then the offset due to cross-talk will have opposite (negative) sign.

A related manifestation of this cross-talk can be seen in some measurements of MCD vs temperature, where the MCD signal can appear to increase in magnitude slowly and linearly as the sample cools from 38 K (T_{N1}) down to 26.5 K (T_{N2}). A discussed above, this is simply a consequence of the emerging linear dichroism due to single-$\mathbf{Q}$ AFM order, causing a small offset in the measurement of circular dichroism. An example of this behavior can be seen in the MCD(T) data shown in Fig. S2c.

C. Mapping out the magnetic phase diagram of $Co_{1/3}TaS_2$: Temperature-dependent MLD(H)

To map out the magnetic phase diagram of $Co_{1/3}TaS_2$, we measured not only H-dependent MCD and MLD

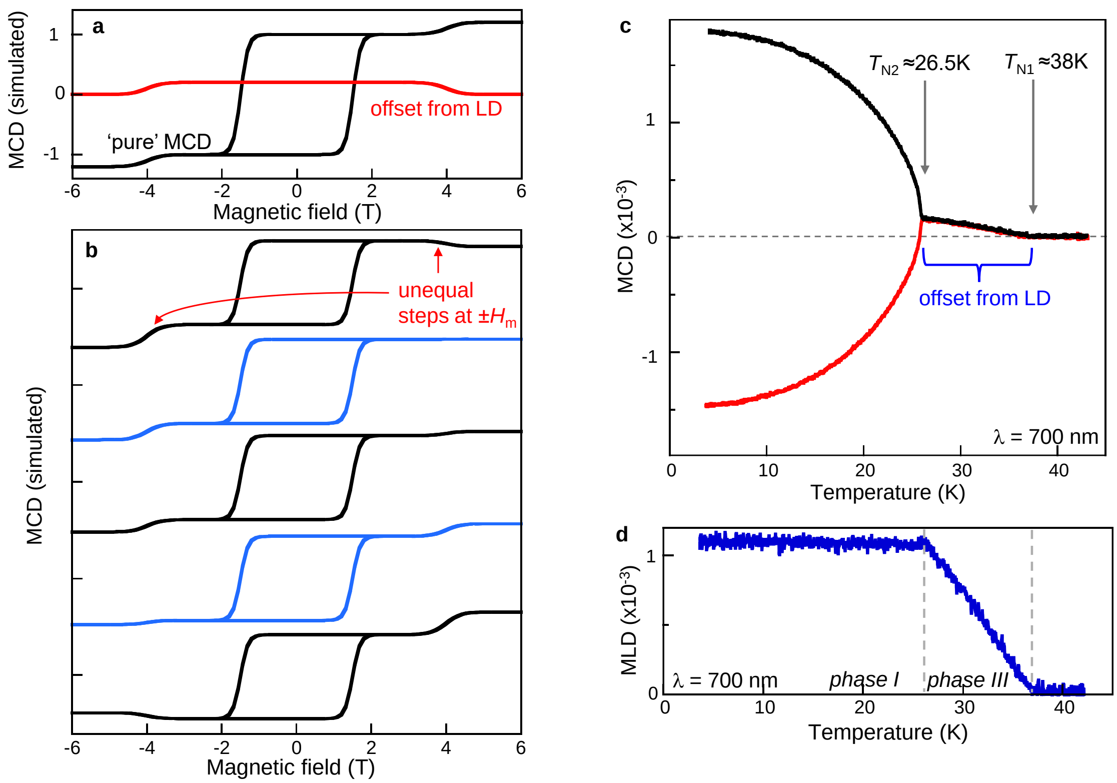

FIG. S2. **Cross-talk between MCD and MLD signals.** The presence of linear dichroism in a sample (due, e.g., to the emergence of nematic order) can lead to offsets (artifacts) in measurements of circular dichroism. **a**, Black trace: a simulated "pure" MCD(H) signal from chiral triple-**Q** AFM order in $Co_{1/3}TaS_2$, showing equal changes at the metamagnetic transitions at $\pm H_m$ (= ± 4 T in these simulations). Red trace: A small offset due to linear dichroism (simulated), that appears when $|H| < H_m$. **b**, Adding or subtracting various amounts of this offset to the pure MCD(H) curve results in MCD(H) traces that have *unequal* step sizes at $H = \pm H_m$, as experimentally observed (*cf.* Figure S1). **c**, Cross-talk between MCD and MLD signals can also be seen in some MCD(T) data, as a small linear change in the MCD signal in the temperature range between 38 K and 26.5 K (*i.e.*, in Phase III), where only single-**Q** nematic order (and therefore only linear dichroism) emerges in $Co_{1/3}TaS_2$. The black and red curves were acquired while warming from $T = 4 \rightarrow 43$ K in H=0, after cooling to T=4 K in $H = \pm 0.5$ T respectively (which poles the chirality of the distorted triple-**Q** AFM order in Phase I). **d**, For reference, the measured MLD(T) at the same location is shown, showing the emergence of linear dichroism due to single-**Q** AFM order upon cooling from 38 K to 26.5 K

curves at different fixed temperatures (shown in Fig. 2 of the main text), but also measured temperature-dependent MCD and MLD curves at different fixed H. Effectively, these represent horizontal line cuts through the (H,T) phase diagram shown in Fig. 2 of the main text.

Examples of MLD(T) curves are shown in Fig. S3, acquired while slowly cooling the sample from approximately 40 → 4 K, at H=0, +1, +2.5, +4, and +6.5 T. As described in the figure caption, these data help to identify phase boundaries at which the single-**Q** nematic (stripe) order changes. Especially noteworthy is the MLD(T) curve acquired at H=+4 T, where the sample goes through all four magnetic phases of $Co_{1/3}TaS_2$ as it cools (paramagnetic → IV → III → I → II).

D. Additional MLD images and analysis of single-Q domains

Figure S4 shows a further analysis of the MLD signals and images that were presented in Fig. 4 of the main text. Fig. S4b shows the nematic director field superimposed on an MLD image (same as Fig. 4d of the main text), with three specific locations indicated. Fig. S4c shows temperature-dependent MLD scans at location #2 (where the nematic director field is oriented along an angle $\theta_{1Q} \approx 120°$), using probe light that is linearly polarized along $\phi = 0°, 120°$, and $240°$. When $\phi = 120°$, the MLD signal increases by a large amount in the positive direction upon cooling from the nonmagnetic state (~40 K) into the single-**Q** AFM phase III (similar, e.g., to Fig. 1g in the main text). This is because ϕ is aligned along the nematic director θ_{1Q} at this location. In contrast, repeating this temperature-dependent MLD scan using light polarized along $\phi = 0°$ or $240°$,

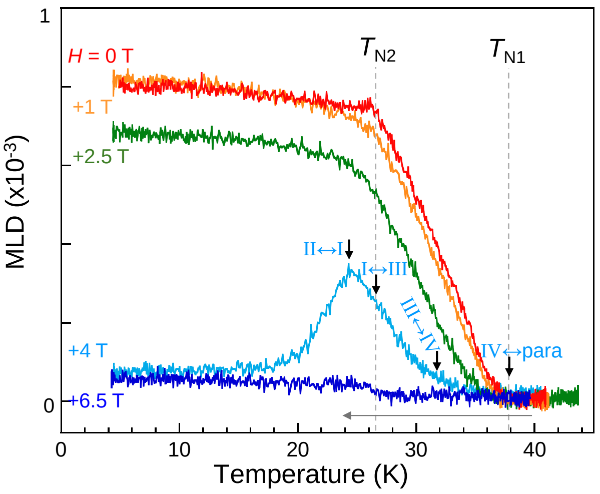

FIG. S3. **Temperature-dependent MLD at different applied fields H.** MLD(T) data help to map out the boundaries of the $Co_{1/3}TaS_2$ magnetic phase diagram, and in particular to identify transitions between phases with different nematic order. At H=0 T (red curve), the transition from the paramagnetic state to the single-**Q** nematic (stripe) phase III is evident at T_{N1}, as is the abrupt saturation of the MLD signal upon further cooling into phase I at T_{N2}. In contrast, at H=+6.5 T (dark blue), the MLD remains approximately zero throughout cooling, indicating that both high-field phases II and IV lack nematic order. Notably, cooling at H=+4T (light blue curve) crosses all four magnetic phases IV→III→I→II, which highlights the utility of the continuous multi-**Q** framework. All data taken using 650 nm probe light.

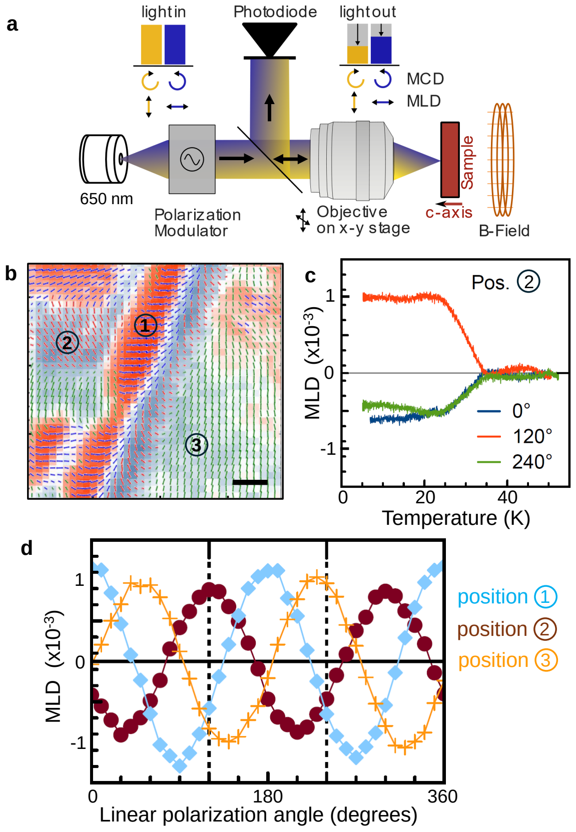

FIG. S4. **Characterizing single-Q AFM domains with MLD microscopy**. **a**, Schematic of the microscopy setup. **b**, Image of nematic director field superimposed on an MLD image taken using probe light linearly polarized along $\phi = 0°$ (same image as Fig 4d of main text). **c**, Temperature-dependent MLD taken at position 2 (where the nematic director is oriented primarily along 120°), using probe light linearly polarized along $\phi = 0°, 120°, 240°$. **d**, MLD signals taken at the three positions indicated, versus the probe light's linear polarization angle ϕ. To remove background signals that are independent of magnetism, these data are the *difference* of MLD(ϕ) scans taken in the single-**Q** state ($\approx$27 K) and in the nonmagnetic state ($\approx$38 K). These data were used to construct the polar plots shown in Fig. 4b of the main text.

the MLD increases in the *negative* direction, but only by about half the amount. This is in line with, and validates, the expectation that MLD signals should scale as $1/2 - \sin^2(\phi - \theta_{1Q})$.

Figure S4d shows measurements of MLD versus ϕ at each of the three locations indicated. Each shows a $\sin(2\phi)$ dependence, as expected for MLD, but shifted in phase by $\pm 120°$. These data were used to construct the polar plots shown in Fig. 4 of the main text.

Additional MLD images were also acquired over a larger area of the sample surface, spanning nearly 1 mm × 1 mm. Figure S5a shows a composite image of the reflected intensity of the probe light used to measure the MLD, over several rectangular regions of the sample surface. From these images, slight variations in the reflected intensity can be observed, revealing what are likely structural imperfections and faults. The corresponding MLD images are shown in Fig. S5b, where it is readily seen that MLD domains are often co-located with the structural imperfections, suggesting that local strain fields likely play a role in determining the patterns of single-**Q** AFM domains. These MLD images were acquired at 8 K, and were not corrected for any background linear dichroism in the paramagnetic state (that is, images acquired at $T > T_{N1}$ were not subtracted).

Figures S5c and S5d show high-resolution images of the measured MLD and associated nematic director fields at two other regions of the $Co_{1/3}TaS_2$ sample surface (indicated by black rectangles in Fig. S5b). As discussed in the main text, the nematic domains are large (many tens of microns) and are found to align primarily along the 0°, 120°, and 240° directions.

2. SUPPLEMENTARY SECTION 2: THEORETICAL MODEL FOR THE CONTINUOUS MULTI-Q MANIFOLD OF M-ORDERING

In this Section, we present a comprehensive theoretical model that describes the general M-orderings on a trian-

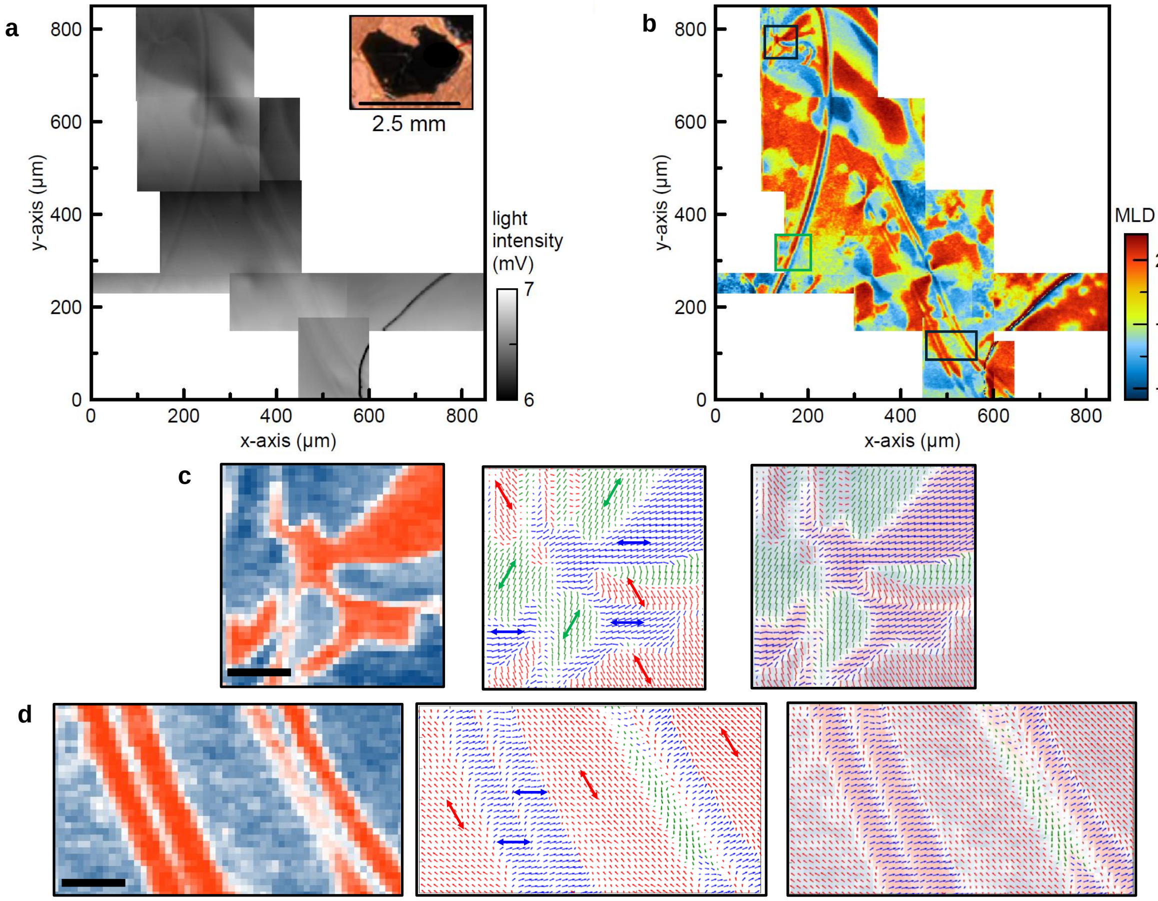

FIG. S5. **Spatially-resolved MLD images over a larger area**. **a**, A composite image showing the intensity of the reflected probe light used in MLD imaging studies, over different regions on the $Co_{1/3}TaS_2$ sample surface, from which surface imperfections can be seen (note: a slow linear drift of the reflected intensity within each sub-image was subtracted). **b**, The corresponding image of the MLD signal. Comparison with panel **a** suggests that single-**Q** AFM domains may be pinned by structural features and faults, and associated local strain fields. **c**, 70×70 μm MLD image of a different region of the $Co_{1/3}TaS_2$ sample, indicated by the black square in panel **b**, acquired using linearly-polarized light modulated between 0° and 90°. The measured nematic director map is shown to the right, and on the far right is the director map superimposed on the MLD image. **d**, Same, but for a different 110×60 μm region, indicated by the black rectangle in panel **b**. Note: The green square in panel **b** indicates the region imaged in Fig. 4 of the main text. All data taken using 650 nm probe light. Scale bars are 20 μm.

gular lattice, i.e., antiferromagnetic orderings where the ordering wave vectors $\mathbf{Q}_\nu (\nu = 1, 2, 3)$ equal half the reciprocal lattice wave vectors and therefore correspond to the M points of the hexagonal Brillouin zone. To capture the rich phase diagram suggested in Fig. 2c, it is essential to consider both four-spin interactions and magnetic anisotropy, in addition to bilinear Heisenberg interaction terms. We elaborate on the role of each term in activating the continuous multi-**Q** manifold and show how the resultant spin model accounts for key experimental observations in $Co_{1/3}TaS_2$.

Our mathematical formulation is based on the real and reciprocal space coordinates shown in Fig. S6a, which leads to $\mathbf{Q}_1 = \mathbf{a}^*/2$, $\mathbf{Q}_2 = -\mathbf{a}^*/2 + \mathbf{b}^*/2$, and $\mathbf{Q}_3 = -\mathbf{b}^*/2$. In other words, $\mathbf{Q}_\nu = \mathbf{G}_\nu/2$ with $\nu = 1, 2, 3$, where $\mathbf{G}_\nu$ are reciprocal lattice vectors related by three-fold rotation about the c-axis.

To describe antiferromagnetic orderings characterized by specific ordering wave vectors, it is convenient to express the spin Hamiltonian in momentum space. We begin with the most general isotropic Hamiltonian that includes bilinear Heisenberg and higher-order four-spin interactions:

$$\hat{\mathcal{H}} = \hat{\mathcal{H}}_{\text{Heis}} + \hat{\mathcal{H}}_{\text{K}} \tag{S1}$$

with

$$\hat{\mathcal{H}}_{\text{Heis}} = \sum_{\mathbf{q}} \tilde{J}^{ab}_{\mathbf{q}} \tilde{\mathbf{S}}^a_{\mathbf{q}} \cdot \tilde{\mathbf{S}}^b_{-\mathbf{q}}$$

$$\hat{\mathcal{H}}_{\text{K}} = \sum_{\mathbf{q}_i, \mathbf{q}_j, \mathbf{q}_k} \frac{\tilde{K}^{abcd}_{\mathbf{q}_i \mathbf{q}_j \mathbf{q}_k}}{N} (\tilde{\mathbf{S}}^a_{\mathbf{q}_i} \cdot \tilde{\mathbf{S}}^b_{\mathbf{q}_j})(\tilde{\mathbf{S}}^c_{\mathbf{q}_k} \cdot \tilde{\mathbf{S}}^d_{-\mathbf{q}_{\text{tot}}}), \tag{S2}$$

where we are adopting the convention of summation for repeated sublattice indices $a, b, c, d \in \{o, e\}$ with o, e

denoting odd and even layers of two Co sublattices, $\mathbf{q}_{\rm tot} = \mathbf{q}_i + \mathbf{q}_j + \mathbf{q}_k$ and

$$\tilde{\mathbf{S}}^a_{\mathbf{q}} = \frac{1}{\sqrt{N}} \sum_{\mathbf{r}} e^{-i\mathbf{q}\cdot\mathbf{r}} \mathbf{S}^a_{\mathbf{r}} \tag{S3}$$

is the Fourier transform of the spin field, where $\mathbf{r}$ spans N crystallographic unit cells of the material of interest.

In this analysis, we assume that $\hat{\mathcal{H}}_{\rm Heis}$ dominates over $\hat{\mathcal{H}}_{\rm K}$, a hierarchy expected to hold in most real materials, including $Co_{1/3}TaS_2$ [3, 4]. Under this assumption, we demonstrate how the general spin model in Eq. (S2) simplifies for the M-ordering problem in $Co_{1/3}TaS_2$.

A. Bilinear Heisenberg interactions and degenerate multi-Q manifold

The first step in analyzing Eq. (S2) is to realize M-ordering by restricting the bilinear Heisenberg interactions so that they favor magnetic ordering wave vectors $\mathbf{Q}_\nu = \mathbf{G}_\nu/2$ [Fig. S6a]. For isotropic Heisenberg interactions described by $\hat{\mathcal{H}}_{\rm Heis}$ this implies that $\tilde{J}_{\mathbf{q}}$ has global minima at $\mathbf{q} = \mathbf{Q}_\nu$ with $\nu = 1, 2, 3$ (see Fig. S6a). When this condition holds, the classical ground state of the system has nonzero Fourier components $\tilde{\mathbf{S}}_{\mathbf{q}}$ only at $\mathbf{q} = \mathbf{Q}_\nu$. Additionally, a finite Fourier component at $\mathbf{q} = \mathbf{0}$–representing non-zero magnetization–is expected to be induced by a uniform magnetic field.

The most general real-space spin configuration that minimizes $\hat{\mathcal{H}}_{\rm Heis}$ described above can be expressed as

$$\mathbf{S}^a(\mathbf{r}) = \sum_\nu \tilde{\mathbf{S}}^a_{\mathbf{Q}_\nu} \cos(\mathbf{Q}_\nu \cdot \mathbf{r}), \tag{S4}$$

where $\mathbf{r}$ is a lattice vector on the two-dimensional triangular lattice; the out-of-plane component of the ordering wave vectors $\mathbf{Q}_\nu$ is equal to zero [Fig. S6a].

For the two Co sublattices arranged in a hexagonal close-packed structure [see Fig. 1a], the antiferromagnetic exchange interactions between even and odd layers [3, 4] ensure that the spin configuration in one layer matches the other when shifted by the vector $\mathbf{t} = (1, 1, 1/2)$ in lattice units. Consequently, the vector amplitudes for the even and odd sublattices are connected by the following simple relation:

$$\tilde{\mathbf{S}}^o_{\mathbf{Q}_\nu} = e^{i\mathbf{Q}_\nu\cdot\mathbf{t}}\, \tilde{\mathbf{S}}^e_{\mathbf{Q}_\nu}. \tag{S5}$$

Given this relationship, in the following we will drop the sublattice index: $\tilde{\mathbf{S}}^o_{\mathbf{Q}_\nu} \equiv \tilde{\mathbf{S}}_{\mathbf{Q}_\nu}$ and $\tilde{\mathbf{S}}^e_{\mathbf{Q}_\nu} \equiv e^{i\mathbf{Q}_\nu\cdot\mathbf{t}}\tilde{\mathbf{S}}_{\mathbf{Q}_\nu}$. In other words, the identical spin configurations across adjacent Co layers effectively reduce the 3D magnetic model of $Co_{1/3}TaS_2$ to that of a 2D triangular magnetism. We define $|\tilde{\mathbf{S}}^o_{\mathbf{Q}_\nu}| = |\tilde{\mathbf{S}}^e_{\mathbf{Q}_\nu}| \equiv \Delta_\nu$ and will use Δ_ν from hereafter.

Given that the system exhibits the shortest antiferromagnetic periodicity ($\mathbf{Q}_\nu = \mathbf{G}_\nu/2$), Eq. (S4) allows for a maximum of four spin sublattices in real space. Consequently, the problem further reduces to a four-site system with real-space coordinates:

$$\mathbf{r}_1 = \mathbf{0}, \quad \mathbf{r}_2 = \mathbf{a}, \quad \mathbf{r}_3 = \mathbf{b}, \quad \mathbf{r}_4 = \mathbf{a} + \mathbf{b}, \tag{S6}$$

where $\mathbf{a}$ and $\mathbf{b}$ are primitive lattice vectors (see Fig. S6a). The four nonzero Fourier components at $\mathbf{q} = \mathbf{0}$ and $\mathbf{q} = \mathbf{Q}_\nu$ – with the first case corresponding to a ferromagnetic component – are related to the four classical spin vectors at $\mathbf{r}_j$ ($j = 1, 2, 3, 4$) as follows:

$$\begin{aligned}
\tilde{\mathbf{S}}_{\mathbf{0}} &= \frac{\sqrt{N}}{4}(\mathbf{S}_1 + \mathbf{S}_2 + \mathbf{S}_3 + \mathbf{S}_4) \\
\tilde{\mathbf{S}}_{\mathbf{Q}_1} &= \frac{\sqrt{N}}{4}(\mathbf{S}_1 - \mathbf{S}_2 + \mathbf{S}_3 - \mathbf{S}_4) \\
\tilde{\mathbf{S}}_{\mathbf{Q}_2} &= \frac{\sqrt{N}}{4}(\mathbf{S}_1 - \mathbf{S}_2 - \mathbf{S}_3 + \mathbf{S}_4) \\
\tilde{\mathbf{S}}_{\mathbf{Q}_3} &= \frac{\sqrt{N}}{4}(\mathbf{S}_1 + \mathbf{S}_2 - \mathbf{S}_3 - \mathbf{S}_4),
\end{aligned} \tag{S7}$$

where $\tilde{\mathbf{S}}_{\mathbf{0}}$ is proportional to the net spin magnetization.

In the classical limit, the four spins $\mathbf{S}_i$ have a fixed magnitude, $|\mathbf{S}_i| = S$. While the longitudinal spin stiffness remains finite in magnetic systems with sizable quantum spin fluctuations (which usually manifest in small-spin systems), this classical approximation should adequately describe the ground-state ordering and its long-wavelength spin fluctuations in $Co_{1/3}TaS_2$, where $S = 3/2$ arises from the d^7 high-spin configuration of Co^{2+}. For compensated antiferromagnetic configurations, where $\tilde{\mathbf{S}}_{\mathbf{0}} = \mathbf{0}$, this spin-length constraint is satisfied when the three vector Fourier components $\mathbf{S}_{\mathbf{Q}_\nu}$ are mutually orthogonal,

$$\tilde{\mathbf{S}}_{\mathbf{Q}_\nu} \perp \tilde{\mathbf{S}}_{\mathbf{Q}_{\nu'}} \ \text{ for } \ \nu \neq \nu' \tag{S8}$$

and satisfy the normalization constraint:

$$\sum_{\nu=1}^{3} \tilde{\mathbf{S}}_{\mathbf{Q}_\nu} \cdot \tilde{\mathbf{S}}_{\mathbf{Q}_\nu} = \sum_\nu {\Delta_\nu}^2 = NS^2. \tag{S9}$$

Under the constraints of Eq. (S8) and (S9), all multi-$\mathbf{Q}$ spin configurations generated by Eq. (S4) are confined to a spherical surface spanned by the three orthogonal basis vectors $\tilde{\mathbf{S}}_{\mathbf{Q}_\nu}$. As illustrated in Fig. 3a or Fig. S6b, this schematic in the $\Delta_1 - \Delta_2 - \Delta_3$ phase space provides a clear representation of the multi-$\mathbf{Q}$ manifold explored in this work. By varying the relative magnitudes of Δ_ν, one can access all possible single-, double-, and triple-$\mathbf{Q}$ spin configurations corresponding to the M-ordering.

Figs. S6b–c show two representative high-symmetry paths on this multi-$\mathbf{Q}$ manifold, describing the continuous interpolation between the most symmetric triple-$\mathbf{Q}$ ordering and the single-$\mathbf{Q}$ ordering (green or orange/purple arrows). The state with $\Delta_1 = \Delta_2 = \Delta_3$ corresponds to the three-fold symmetric triple-$\mathbf{Q}$ ordering,

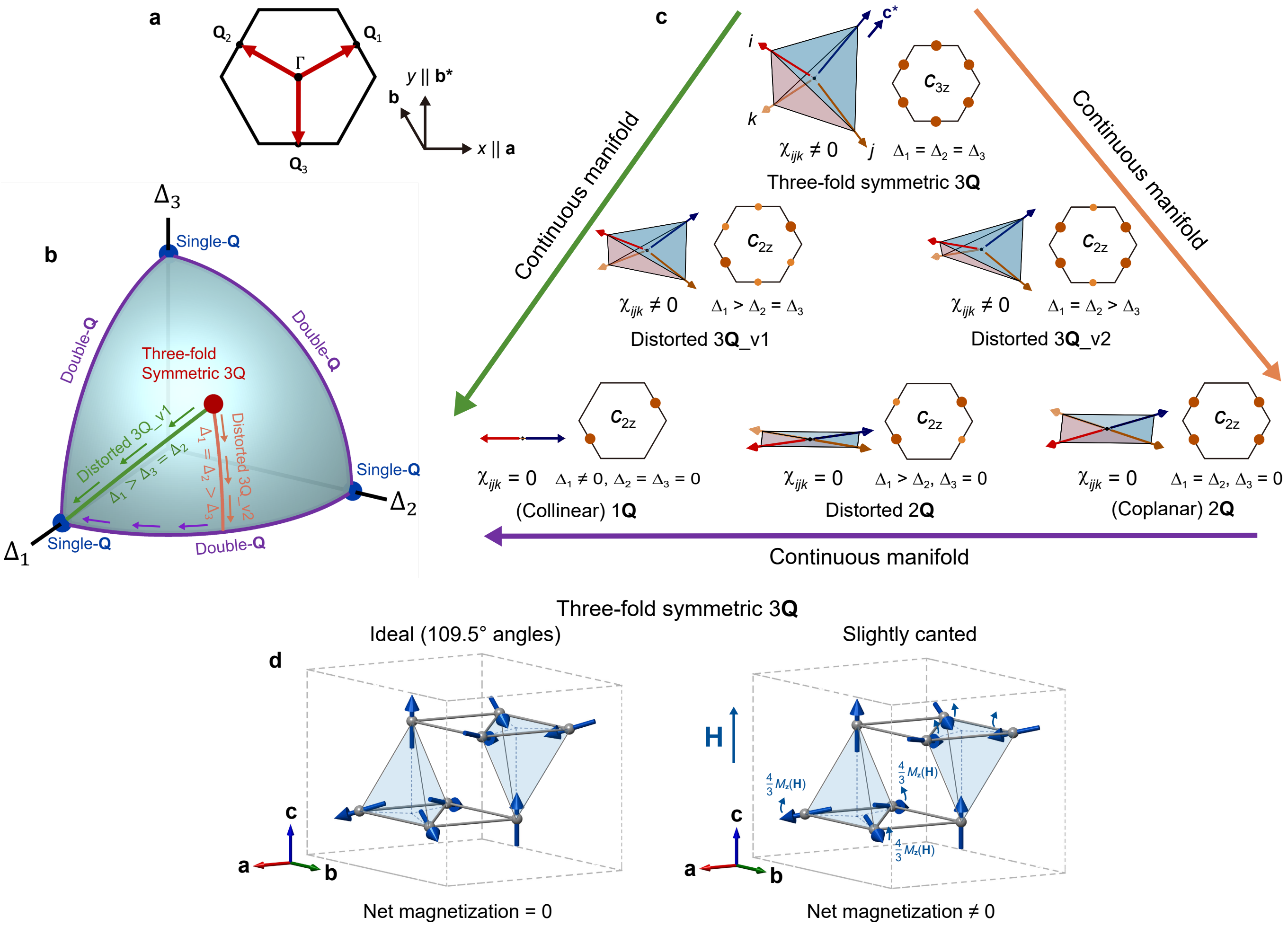

FIG. S6. **Continuous manifold of multi-Q M-ordering spanned by Eq. (S4).** **a**, Real and reciprocal lattice coordinate conventions referenced in this study. The three red arrows denote the ordering wave vectors of the M-ordering, $\mathbf{Q}_\nu$ (ν=1,2,3). **b**, Continuous manifold of multi-**Q** M-orderings, shown as a variational space on a spherical shell. Same as Fig. 3a but with an additional highlight of the orange path connecting the three-fold symmetric triple-**Q** and double-**Q** orderings. **c**, Magnitude of the Fourier components (Δ_ν) and the corresponding real-space configurations of the four spin sublattices for various phases on the manifold. **d**, Two possible configurations of the three-fold symmetric triple-**Q** state. The left panel illustrates an ideal four-sublattice structure forming an equilateral tetrahedron with mutual angles of 109.5° and zero net magnetization. The right panel shows a modified configuration that still satisfies $\Delta_1 = \Delta_2 = \Delta_3$, but includes a small net out-of-plane magnetization and therefore slight spin canting –induced by an external magnetic field or magnetic anisotropy– which distorts the spin angles away from exactly 109.5°. The latter configuration is found in Phase II.

where the four spin sublattices individually align with the principal directions of a regular tetrahedron. Notably, this is the only configuration that preserves the hexagonal rotation symmetry of the triangular lattice. However, when $\Delta_1 = \Delta_2$ but $\Delta_3 \neq \Delta_1$, the tetrahedron spanning the four spin sublattices becomes distorted (labeled "distorted 3Q_v1" or "distorted 3Q_v2" in Fig. S6c). These intermediate states smoothly connect the three-fold symmetric triple-**Q** ordering to either a single-**Q** or double-**Q** ordering. Importantly, these intermediate states exhibit two-fold rotational symmetry (C_{2z}), similar to single-**Q** or double-**Q** orderings, while retaining the net scalar spin chirality of the three-fold symmetric triple-**Q** ordering, consistent with the symmetry argument for Phase I in Fig. 2c.

It is important to emphasize that, under the constraints of Eq. (S8) and (S9), and given that $\mathbf{Q}_\nu = -\mathbf{Q}_\nu$, the continuous multi-**Q** manifold spanned by Eq. (S4) remains fully degenerate when $\hat{\mathcal{H}} = \hat{\mathcal{H}}_{\rm Heis}$. Additionally, we stress that magnetic anisotropy alone cannot induce a multi-**Q** ground state, as will be discussed in the following subsections. Therefore, the four-spin term $\hat{\mathcal{H}}_{\rm K}$ is essential for realizing four-sublattice multi-**Q** magnetic ground states, as also highlighted in Refs. [3–5].

B. Four-spin interactions

The accidental multi-$\mathbf{Q}$ degeneracy described earlier is lifted by $\hat{\mathcal{H}}_{\mathrm{K}}$. As noted in the introduction to Section 2, $\hat{\mathcal{H}}_{\mathrm{K}}$ is much weaker than $\hat{\mathcal{H}}_{\mathrm{Heis}}$, ensuring that it does not generate any additional Fourier components beyond the four $\tilde{\mathbf{S}}_{\mathbf{Q}_\nu}(\nu = 0,1,2,3)$ in Eq. (S7). Thus, by using Eq. (S5), the classical energy due to four-spin interactions [$\hat{\mathcal{H}}_{\mathrm{K}}$ in Eq. (S2)] reduces to [6]:

$$E_{\mathrm{K}} = \frac{\tilde{K}_1}{2N}\sum_{\nu\neq\nu'}\left(\tilde{\mathbf{S}}_{\mathbf{Q}_\nu}\cdot\tilde{\mathbf{S}}_{\mathbf{Q}_{\nu'}}\right)^2 + \frac{\tilde{K}_2}{N}\sum_{\nu=1}^{3}\left(\tilde{\mathbf{S}}_{\mathbf{Q}_\nu}\cdot\tilde{\mathbf{S}}_{\mathbf{Q}_\nu}\right)^2 + \frac{\tilde{K}_3}{2N}\sum_{\nu\neq\nu'}\left(\tilde{\mathbf{S}}_{\mathbf{Q}_\nu}\cdot\tilde{\mathbf{S}}_{\mathbf{Q}_\nu}\right)\left(\tilde{\mathbf{S}}_{\mathbf{Q}_{\nu'}}\cdot\tilde{\mathbf{S}}_{\mathbf{Q}_{\nu'}}\right) \quad \text{(S10)}$$

The first term vanishes ($E_1 = 0$) because of the orthogonality condition given in Eq. (S8). In addition, Eq. (S9) implies that E_2 and E_3 are connected by a simple linear relation

$$E_2 = -2E_3 + NS^4, \quad \text{(S11)}$$

implying that $\langle\mathcal{H}_{\mathrm{K}}\rangle$ depends on a single coefficient:

$$E_{\mathrm{K}} = (\tilde{K}_2 - \frac{\tilde{K}_3}{2})E_2 + \frac{\tilde{K}_3 S^4}{2} \equiv \tilde{K}E_2 + \text{const}, \quad \text{(S12)}$$

with

$$E_2 = \frac{1}{N}\langle\sum_{\nu=1}^{3}\left(\tilde{\mathbf{S}}_{\mathbf{Q}_\nu}\cdot\tilde{\mathbf{S}}_{\mathbf{Q}_\nu}\right)^2\rangle = \frac{1}{N}({\Delta_1}^4 + {\Delta_2}^4 + {\Delta_3}^4). \quad \text{(S13)}$$

Consequently, the combination of $\mathcal{H}_{\mathrm{Heis}}$ and $\mathcal{H}_{\mathrm{K}}$ can produce only two possible magnetic ground states: (1) a three-fold symmetric triple-$\mathbf{Q}$ magnetic ordering ($\Delta_1 = \Delta_2 = \Delta_3$) when $\tilde{K} > 0$, and (2) a stripe single-$\mathbf{Q}$ ordering ($\Delta_1 = \sqrt{N}S$ and $\Delta_2 = \Delta_3 = 0$) when $\tilde{K} < 0$. The colour plot in Fig. S7a shows E_2 in the $\Delta_1 - \Delta_2 - \Delta_3$ space, illustrating the energy landscape for $\tilde{K} > 0$, which indeed favors the three-fold symmetric configuration where $\Delta_1 = \Delta_2 = \Delta_3$.

In $Co_{1/3}TaS_2$, $\tilde{K} > 0$ accounts for the triple-$\mathbf{Q}$ and single-$\mathbf{Q}$ nature of the magnetic orderings in $T < T_{\mathrm{N2}}$ (Phase I) and $T_{\mathrm{N2}} < T < T_{\mathrm{N1}}$ (Phase III), respectively [3, 4]. Although $\tilde{K} > 0$ leads to a triple-$\mathbf{Q}$ ground state at zero temperature, collinear single-$\mathbf{Q}$ ordering can still emerge as a finite-temperature ground state due to the order-by-thermal-disorder mechanism [7, 8]. However, the three-fold symmetric ground state predicted by this generalized four-spin model fails to account for the broken C_{3z} symmetry observed in $T < T_{\mathrm{N2}}$ and $H = 0$ through our MLD measurements. This discrepancy motivates the inclusion of magnetic anisotropy in our model, which is supported by several experimental observations in $Co_{1/3}TaS_2$ (see Section 2D).

C. Equivalence to the real-space scalar biquadratic interaction model

Before including magnetic anisotropy, we discuss the correspondence between the Fourier transformed $\hat{\mathcal{H}}_{\mathrm{K}}$, simplified into Eq. (S12), and the simplest real-space scalar biquadratic interaction term ($\hat{\mathcal{H}}_{\mathrm{bq}}$) between nearest-neighbors (NNs):

$$\hat{\mathcal{H}}_{\mathrm{bq}} = K\sum_{\mathbf{r},\boldsymbol{\delta}_1}(\hat{\mathbf{S}}_{\mathbf{r}}\cdot\hat{\mathbf{S}}_{\mathbf{r}+\boldsymbol{\delta}_1})^2, \quad \text{(S14)}$$

where $\boldsymbol{\delta}_1$ runs over the bond vectors connecting NNs in a triangular lattice, without double-counting. As far as the bilinear Heisenberg interactions are much stronger than K and produce M-ordering, the most general magnetic ordering is still a four-sublattice structure, as dictated by Eq. (S7). An explicit form of Eq. (S14) based on these four spin sublattices is obtained as follows:

$$E_{\mathrm{bq}} = \frac{K}{N}\sum_{j\neq j'}(\mathbf{S}_j\cdot\mathbf{S}_{j'})^2 \quad \text{(S15)}$$

with $j, j' = 1,2,3,4$.

Replacing $\mathbf{S}_i$ in Eq. (S15) with $\tilde{\mathbf{S}}_{\mathbf{Q}_\nu}$ (assuming $|\tilde{\mathbf{S}}_{\mathbf{0}}| \ll S$) yields:

$$E_{\mathrm{bq}} = \frac{2K}{N}\Big[6\sum_{\nu=1}^{3}\Delta_\nu^4 - \sum_{\nu\neq\nu'}\Delta_\nu^2\Delta_{\nu'}^2\Big]. \quad \text{(S16)}$$

Using Eq. (S9), it can be shown that the sum of the last three terms is proportional to the first term, up to an additive constant. This results in the following simplified expression:

$$E_{\mathrm{bq}} = 14\frac{K}{N}\Big[\sum_{\nu=1}^{3}\Delta_\nu^4 - \frac{N^2S^4}{7}\Big] = 14KE_2 + \text{const.} \quad \text{(S17)}$$

The E_{bq} term precisely coincides with the simplified form of the Fourier transformed E_{K} derived in the previous subsection, except for a different constant and scaling factor, which do not alter the overall energy landscape. Thus, E_{bq} produces the same classical energy landscape as E_{K} shown in Fig. S7a, implying that a bi-quadratic interaction is enough to model the most general classical ground state of the 4-sublattice structure and its long-wavelength (i.e., low-energy) fluctuations.

This equivalence is particularly valuable for numerical spin simulations. In $Co_{1/3}TaS_2$, incorporating thermal fluctuations is essential for realizing Phase III or Phase IV, for which classical Monte Carlo simulations have proven highly effective [3, 4]. However, classical Monte Carlo simulations can only be performed with a real-space spin Hamiltonian. The equivalence demonstrated in this subsection ensures that we can accurately simulate

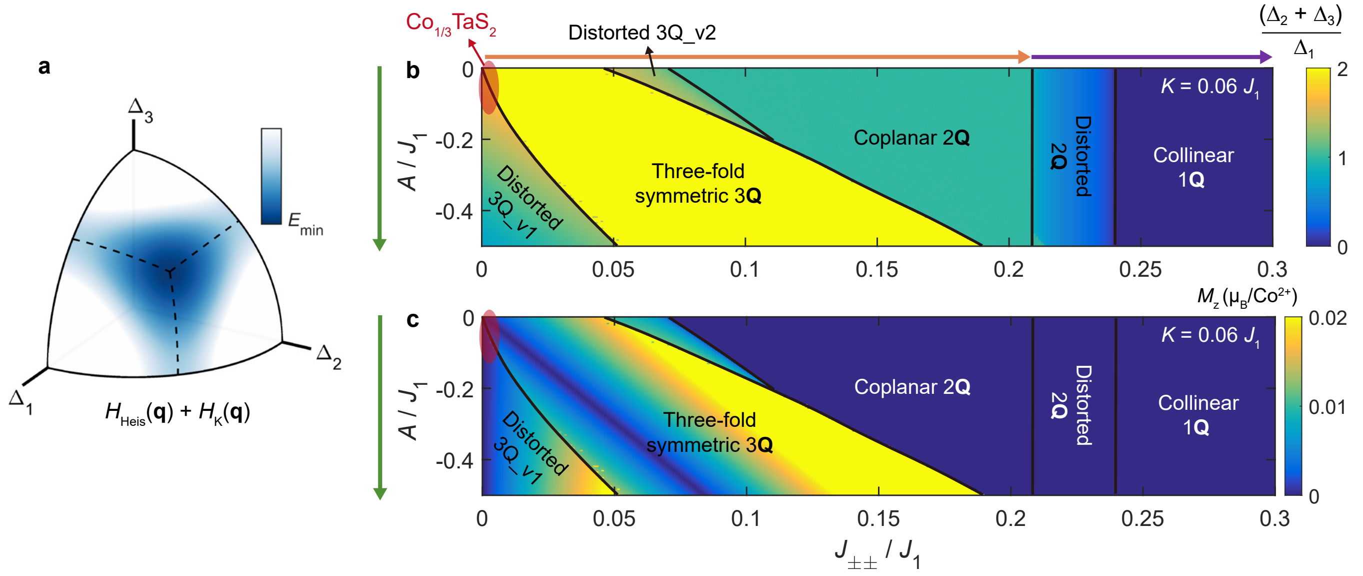

FIG. S7. **Theoretical zero-temperature phase diagram of the isotropic and anisotropic spin Hamiltonian.** **a**, Classical energy landscape of the isotropic spin Hamiltonian (Eq. (S2)), indicating a three-fold symmetric triple-**Q** ground state for $\tilde{K} > 0$. Note that the Heisenberg term yields a constant energy across the entire variation space, making the landscape solely dependent on the four-spin interaction term (Eq. (S12)). **b**–**c**, Phase diagram spanned by single-ion anisotropy A and bond-dependent exchange anisotropy $J_{\pm\pm}$, in the presence of $K = 0.06J_1$. The colour scales in **b** and **c** represent $\frac{\Delta_2+\Delta_3}{\Delta_1}$ ($\Delta_3 \leq \Delta_2 \leq \Delta_1$) and the out-of-plane net magnetization (M_z), respectively, as obtained from our ground state search algorithm (see Supplementary Section 2E). The spin configurations of each phase are shown in Fig. S6c and Fig. S9.

the low-energy physics of the generalized momentum-space Hamiltonian using the real-space Hamiltonian, which includes Heisenberg interaction terms and the nearest-neighbor (NN) scalar bi-quadratic term $\hat{\mathcal{H}}_{\mathrm{bq}}$. However, while this model can describe the ground state and low-energy modes, it should not be considered a microscopic Hamiltonian for $Co_{1/3}TaS_2$. The underlying physics of long-range exchange interactions in metallic $Co_{1/3}TaS_2$ is more accurately captured by the Fourier-transformed form $\hat{\mathcal{H}}_{\mathrm{K}}$, which avoids introducing any artificial cut-off in the real-space exchange interactions.

The effective bilinear and biquadratic exchange constants of the real-space spin Hamiltonian were determined in previous inelastic neutron scattering (INS) studies. Notably, Ref. [4] proposed a reliable model that accounts for both intralayer and interlayer couplings up to a bond length of 11.5 Å. This model successfully describes the low-energy spin dynamics of $Co_{1/3}TaS_2$ in both Phase I and Phase III, as well as the full spin dynamics in the paramagnetic phase ($T > T_{\mathrm{N1}}$). All classical Monte Carlo simulations and zero-temperature phase diagrams were computed using the bilinear exchange coefficients and K suggested in Ref. [4], along with the magnetic anisotropy terms introduced in Section 2D below.

D. Magnetic anisotropy

In this subsection, we introduce two types of magnetic anisotropy present in $Co_{1/3}TaS_2$, which are crucial for explaining our observations from magneto-optical measurements. Generally, easy-axis magnetic anisotropy favors collinear single-**Q** magnetic ordering, as shown in Figs. 3e and S6c. When the anisotropy is much weaker than the four-spin interactions, the system retains the three-fold symmetric triple-**Q** ordering described in Section 2B. However, when both perturbations are comparable—our focus in this work—the competition between them can result in intermediate states that interpolate between the three-fold symmetric triple-**Q** and single-**Q** orderings. We demonstrate that this competition can lead to a distorted triple-**Q** ground state, which is consistent with the symmetry and chirality features of Phase I suggested by our MLD and MCD measurements.

The primary term of interest is the single-ion easy-axis anisotropy:

$$\hat{\mathcal{H}}_{\mathrm{SI}} = A \sum_{\mathbf{r}} (\hat{S}_{\mathbf{r}}^{z,a})^2, \qquad \text{(S18)}$$

where $A < 0$ and $S_{\mathbf{r}}^{z,a}$ is the out-of-plane component of the spin vector at unit cell $\mathbf{r}$ and sublattice a. Phase III in Fig. 2c suggests the presence of this term. Previous neutron diffraction studies have shown that this single-**Q** state comprises only out-of-plane components [3, 9] (see Fig. 3e), indicating that the c-axis is the easy-axis for the magnetic moments in $Co_{1/3}TaS_2$. This anisotropy can

alternatively be described by introducing XXZ exchange anisotropy in the bilinear interactions, which produces the same effect as $\hat{\mathcal{H}}_{\rm SI}$.

The second source of magnetic anisotropy is $J_{\pm\pm}$, which, along with $J_{z\pm}$, represents the symmetry-allowed bond-dependent anisotropic exchange terms (see Ref. [10]):

$$\hat{\mathcal{H}}_{\pm\pm} = \sum_{\langle i,j\rangle_1} 2J_{\pm\pm}\Big[\left(S_i^x S_j^x - S_i^y S_j^y\right)\cos\phi_\alpha - \left(S_i^x S_j^y + S_i^y S_j^x\right)\sin\phi_\alpha\Big] \quad \text{(S19)}$$

where $\langle i,j\rangle_1$ runs over the bonds between nearest neighbours, $x \parallel a$-axis, and $\phi_\alpha \in \{0, 2\pi/3, 4\pi/3\}$ is the angle between a bond vector ($i \to j$) and the a-axis (i.e., bond-dependent). Similar to $\hat{\mathcal{H}}_{\rm SI}$, the presence of a finite $J_{\pm\pm} > 0$ is supported by previous experimental observations. First, the $J_{\pm\pm}(> 0)$ term aligns all four spins along high-symmetry crystalline directions by breaking continuous spin-rotational symmetry [11], as suggested by Rietveld refinement analysis from previous neutron diffraction studies [3, 9]. This broken spin-rotational symmetry accounts for the small energy gap of the Goldstone magnon mode in the triple-$\mathbf{Q}$ phase of $\mathrm{Co_{1/3}TaS_2}$ [3]. Second, it induces a slight out-of-plane spin canting in tetrahedral triple-$\mathbf{Q}$ orderings (both three-fold symmetric and distorted), which results in a tiny net magnetization along the c-axis (see Fig. S7c), consistent with experimental observations (Fig. 1d).

It is worth noting that such anisotropy is common in triangular lattice magnets [12]. However, a detailed investigation of the impact of these anisotropic terms on the M-ordering problem ($\mathbf{Q}_\nu = \mathbf{G}_\nu/2$) has yet to be conducted.

While accurately determining the magnitudes of A and $J_{\pm\pm}$ is not feasible with the available data, their upper limits can be reasonably estimated based on previous INS studies [3, 4]. The direct consequence of finite $J_{\pm\pm}$ and A is the opening of an energy gap in the Goldstone magnon mode for the triple-$\mathbf{Q}$ ($T < T_{\rm N2}$) and single-$\mathbf{Q}$ ($T_{\rm N2} < T < T_{\rm N1}$) phases, respectively. In the triple-$\mathbf{Q}$ phase, a small energy gap of approximately 0.5 meV has been observed [3]. A simple spin-wave calculation, including proper resolution convolution effects, suggests that for $J_{\pm\pm}$ values larger than 0.0024 meV (approximately $0.002J_1$), a gap larger than 0.5 meV is opened. Therefore, a reasonable range for $J_{\pm\pm}$ is from 0 to roughly $0.002J_1$.

Estimating A is more complex. Experimentally, no energy gap has been detected in the single-$\mathbf{Q}$ spin-wave spectrum of $T_{\rm N2} < T < T_{\rm N1}$ (Phase III), with a precision down to 0.2 meV [4]. However, it should be noted that this intermediate single-$\mathbf{Q}$ ordering emerges under significant thermal fluctuations via the order-by-thermal-disorder mechanism [7, 8]. As a result, the observed spectrum should not exhibit an energy gap for small enough values of $|A|$. Our preliminary investigation into the relationship between $|A|$ and the energy gap under significant thermal fluctuations, using Landau-Lifshitz dynamics (LLD) simulations within the Su(n)ny software package [13, 14], suggests that the approximate range for A in $\mathrm{Co_{1/3}TaS_2}$ is between 0 and $0.06J_1$, which is smaller than $|K|$.

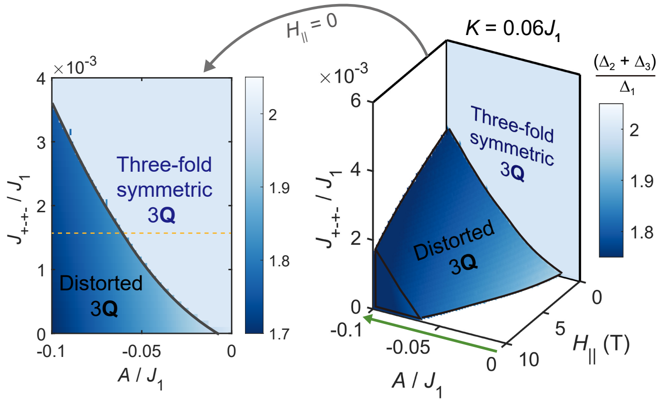

FIG. S8. **Theoretical zero-temperature phase diagram of the anisotropic spin Hamiltonian for $\mathrm{Co_{1/3}TaS_2}$** (Eqs. (S18) and (S19)) under an out-of-plane magnetic field (H). While a non-zero A induces the distorted 3$\mathbf{Q}$ state, H favors the three-fold symmetric 3$\mathbf{Q}$ state. The left panel shows the $H = 0$ slice of the $A - J_{\pm\pm} - H$ phase diagram.

E. Phase diagram spanned by anisotropy

In this subsection, we explain how magnetic anisotropy naturally accounts for the rich phase diagram observed in MLD and MCD measurements (Fig. 2c). Notably, it accurately predicts Phase I by producing a distorted triple-$\mathbf{Q}$ ground state, which breaks three-fold rotational symmetry while preserving nearly the same chiral structure as the three-fold symmetric triple-$\mathbf{Q}$ ordering.

We calculated the $T = 0$ magnetic phase diagram for the spin Hamiltonian with isotropic bilinear and biquadratic interactions, as well as $\hat{\mathcal{H}}_{\rm SI}$ and $\hat{\mathcal{H}}_{\pm\pm}$ discussed above. This was achieved by minimizing the classical energy of the four spin sublattice configuration $\mathbf{S}_j$ ($j = 1,2,3,4$) using the conjugate gradient method implemented in the Su(n)ny software package [13, 14]. The four vector Fourier components in Eq. S7 were subsequently calculated from the optimal real space configuration $\mathbf{S}_j$. From these components, we can classify the ground state orderings as: single-$\mathbf{Q}$ (one non-zero $\Delta_{1,2,3}$), double-$\mathbf{Q}$ (two non-zero $\Delta_{1,2,3}$), distorted triple-$\mathbf{Q}$ (three non-zero $\Delta_{1,2,3}$ but with different magnitudes), and three-fold symmetric triple-$\mathbf{Q}$ (three non-zero $\Delta_{1,2,3}$ with equal magnitudes). These states are effectively visualized using the ratio $(\Delta_2 + \Delta_3)/\Delta_1$ where $\Delta_1 > \Delta_2 > \Delta_3$.

Fig. S7b presents the resultant zero-temperature magnetic phase diagram over a wide range of A and $J_{\pm\pm}$ values, in units of $J_1 = 1.212$ meV [4]. The isotropic in-

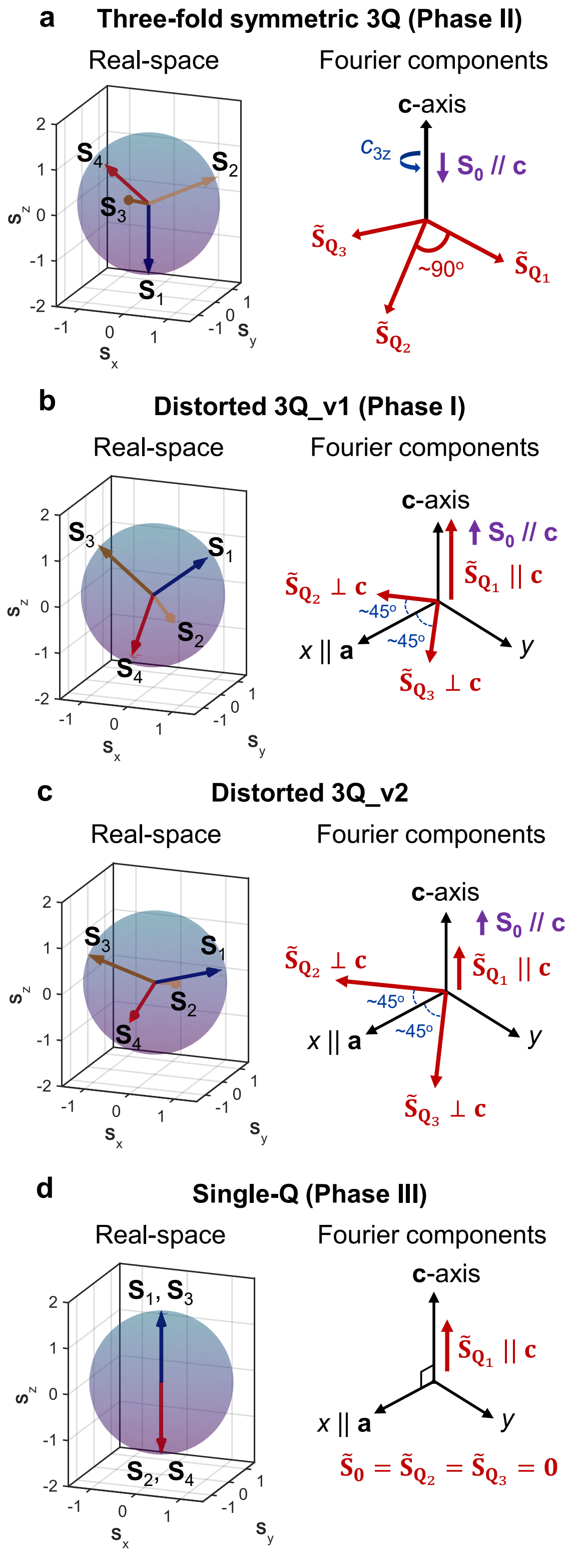

FIG. S9. **Real-space configurations of the four spin sublattices for several multi-Q magnetic ground states predicted by our anisotropic spin Hamiltonian**; see Fig. S7b. The right side of each panel illustrates the corresponding vector Fourier components $\tilde{\mathbf{S}}_{\mathbf{Q}_\nu}$ (ν=1,2,3).

teraction coefficients were adopted from Ref. [4]: $J_1 = 1.212\,\mathrm{meV}$, $J_2 = 0.264J_1$, $J_3 = 0.018J_1$, $J_{c1} = 1.160J_1$, $J_{c2} = -0.215J_1$, and $K = 0.06J_1$, where J_n (J_{cm}) denotes the interaction between n^{th} intralayer (m^{th} interlayer) NN spins. As described in the previous subsection, increasing the strength of each anisotropy gradually transforms the three-fold symmetric triple-**Q** ground state into a single-**Q** state, with a distorted triple-**Q** ground state emerging in the intermediate regime. Notably, increasing $|A|$ and $J_{\pm\pm}$ follows two distinct high-symmetry routes on the continuous multi-**Q** manifold. The path driven by A (shown by green arrows) and the path driven by $J_{\pm\pm}$ (shown by orange and purple arrows) are illustrated throughout Fig. S6b–c and S7b.

Based on the feasible magnitudes of $|A|$ and $J_{\pm\pm}$ discussed in the previous subsection, we have indicated the location of $Co_{1/3}TaS_2$ on the phase diagram (shown as a red ellipsoidal region in Fig. S7b). An enlarged view of this region is displayed in the left panel of Fig. S8. Notably, once A becomes non-zero, the ground state immediately transitions from the three-fold symmetric triple-**Q** state to the distorted triple-**Q** state ("Distorted 3Q_v1" in Fig. S6c). Thus, it is natural for a system with slight easy-axis anisotropy to exhibit distorted triple-**Q** ordering with broken C_{3z} symmetry, which is consistent with our observation of Phase I.

Fig. S9b shows the real-space spin configuration and the corresponding Fourier components of the Distorted 3Q_v1 phase, which we identify as the most likely magnetic structure for Phase I in Fig. 2c. First, the orientations of the three Fourier components differ from those of the three-fold symmetric triple-**Q** ordering (Fig. S9a): while $\tilde{\mathbf{S}}_{\mathbf{Q}_1}$ is parallel to the c-axis, $\tilde{\mathbf{S}}_{\mathbf{Q}_2}$ and $\tilde{\mathbf{S}}_{\mathbf{Q}_3}$ lie within the $a-b$ plane and are separated by $\pm(45°+\delta)$ from the a-axis, where $\delta \ll 1$ depends on the magnitude of the weak ferromagnetic moment along the c-axis (see Fig. S7c). Second, while the two in-plane Fourier components have equal magnitudes, the out-of-plane Fourier component is larger ($\Delta_1 > \Delta_2 = \Delta_3$), placing this phase as an intermediate state between the three-fold symmetric triple-**Q** and single-**Q** orderings. Notably, both of these features break the C_{3z} symmetry in this phase (i.e., the symmetry is reduced to C_{2z}).

For comparison, we also present the Distorted 3Q_v2 phase in Fig. S9c. Although this phase shares the same vector Fourier component configuration as the Distorted 3Q_v1 phase (Fig. S9b), the two in-plane Fourier components have a greater magnitude than the out-of-plane component ($\Delta_1 < \Delta_2 = \Delta_3$), indicating its nature as an intermediate phase between the three-fold symmetric triple-**Q** and double-**Q** states. However, realizing this phase would require an order-of-magnitude larger $J_{\pm\pm}$ than what was estimated based on the spin gap. Therefore, it is unlikely that this phase corresponds to Phase I in $Co_{1/3}TaS_2$.

F. External magnetic field

To study the transition between Phase I and Phase II in Fig. 2c and 3, we additionally incorporated the out-of-plane magnetic field into the spin model via the Zeeman term:

$$\hat{\mathcal{H}}_{\mathrm{z}} = g\mu_{\mathrm{B}} H_{\|} \sum_{\mathbf{r},a} \hat{S}^{z,a}_{\mathbf{r}}, \tag{S20}$$

where $g = 2$ (the orbital magnetic moments are believed to be quenched in $Co_{1/3}TaS_2$) and $H_{||}$ denotes the strength of the out-of-plane field. The full three-dimensional phase diagram, spanned by A, $J_{\pm\pm}$, and $H_{||}$, is shown in the right panel of Fig. S8. Indeed, Eq. (S20) favors the three-fold symmetric triple-**Q** magnetic ordering (Fig. S9a) over the distorted triple-**Q** state (Fig. S9b), thereby restoring the C_{3z} rotational symmetry under a strong out-of-plane magnetic field. This is consistent with our observation that the MLD signal disappears in Phase II when $H > H_{\mathrm{m}}$; see Fig. 2c. The temperature-field phase diagram, obtained by classical Monte Carlo simulations using reasonable values for A and $J_{\pm\pm}$ for $Co_{1/3}TaS_2$ (namely, $A = 0.06J_1$ and $J_{\pm\pm} = 0.0016J_1$) is shown in Fig. 3f–g. As demonstrated, our spin model successfully captures the experimental observations related to Phase I, II, III.

[1] Y. Shindo and M. Nishio, The effect of linear anisotropies on the CD spectrum: Is it true that the oriented polyvinyalcohol film has a magic chiral domain inducing optical activity in achiral molecules?, Biopolymers: Original Research on Biomolecules **30**, 25 (1990).

[2] T. J. Ugras, Y. Yao, and R. D. Robinson, Can we still measure circular dichroism with circular dichroism spectrometers: The dangers of anisotropic artifacts, Chirality **35**, 846 (2023).

[3] P. Park, W. Cho, C. Kim, Y. An, Y.-G. Kang, M. Avdeev, R. Sibille, K. Iida, R. Kajimoto, K. H. Lee, et al., Tetrahedral triple-Q magnetic ordering and large spontaneous hall conductivity in the metallic triangular antiferromagnet $Co_{1/3}TaS_2$, Nature Communications **14**, 8346 (2023).

[4] P. Park, W. Cho, C. Kim, Y. An, K. Iida, R. Kajimoto, S. Matin, S.-S. Zhang, C. D. Batista, and J.-G. Park, Spin dynamics of triple-**Q** magnetic orderings in a triangular lattice: Implications for multi-**Q** orderings in general two-dimensional lattices, Phys. Rev. X **15**, 031032 (2025).

[5] C. D. Batista, S.-Z. Lin, S. Hayami, and Y. Kamiya, Frustration and chiral orderings in correlated electron systems, Reports on Progress in Physics **79**, 084504 (2016).

[6] V. Sharma, Z. Wang, and C. D. Batista, Machine learning assisted derivation of minimal low-energy models for metallic magnets, npj Computational Materials **9**, 192 (2023).

[7] J. Villain, R. Bidaux, J.-P. Carton, and R. Conte, Order as an effect of disorder, Journal de Physique **41**, 1263 (1980).

[8] C. L. Henley, Ordering due to disorder in a frustrated vector antiferromagnet, Physical Review Letters **62**, 2056 (1989).

[9] H. Takagi, R. Takagi, S. Minami, T. Nomoto, K. Ohishi, M.-T. Suzuki, Y. Yanagi, M. Hirayama, N. Khanh, K. Karube, et al., Spontaneous topological Hall effect induced by non-coplanar antiferromagnetic order in intercalated van der Waals materials, Nature Physics **19**, 961 (2023).

[10] P. A. Maksimov, Z. Zhu, S. R. White, and A. L. Chernyshev, Anisotropic-exchange magnets on a triangular lattice: Spin waves, accidental degeneracies, and dual spin liquids, Phys. Rev. X **9**, 021017 (2019).

[11] F. Nickel, A. Kubetzka, S. Haldar, R. Wiesendanger, S. Heinze, and K. von Bergmann, Coupling of the triple-q state to the atomic lattice by anisotropic symmetric exchange, Physical Review B **108**, L180411 (2023).

[12] S. Hayami and Y. Motome, Topological spin crystals by itinerant frustration, Journal of Physics: Condensed Matter **33**, 443001 (2021).

[13] D. Dahlbom, H. Zhang, C. Miles, S. Quinn, A. Niraula, B. Thipe, M. Wilson, S. Matin, H. Mankad, S. Hahn, et al., Sunny. jl: A julia package for spin dynamics, arXiv preprint arXiv:2501.13095 (2025).

[14] Su(n)ny, spin dynamics and generalization to SU(N) coherent states, https://github.com/sunnysuite/sunny.jl.
